# CAREGIVER SINGING VERSUS MUSIC ACTIVITIES IN DEMENTIA CARE: DIFFERENT BENEFITS IN DIFFERENT OCCASIONS

**DOI:** 10.1093/geroni/igac059.2054

**Published:** 2022-12-20

**Authors:** Lena Hammar, Annica Lövenmark, Anna Swall

**Affiliations:** Mälardalen University, Västerås, Vastmanlands Lan, Sweden; Mälardalen University, Västerås, Vastmanlands Lan, Sweden; Dalarna University, Falun, Dalarnas Lan, Sweden

## Abstract

Music has been used in the care of persons with dementia (PWDs) in decades, and research show that music activities such as background music, music listening, sing-along activities and playing instruments have positive effects on mood, emotions and interaction. However, Caregiver Singing (CS)- when caregivers are singing for or together with PWDs during care activities, are commonly in literature mixed up with other music activities. CS is a care intervention aiming to facilitate care situations and has shown to be useful to increase interaction and cooperation in care activities. This study aimed to describe caregivers’ experiences of the differences between using CS compared to music in the care of persons with dementia.Data was three focus group interviews with 12 professional caregivers and residential facilities for PWDs. Data was analyzed with qualitative content analysis. Results revealed that CS are a successful tool to communicate, cooperate and bring out the PWDs hidden resources during care activities. The PWDs were told to be more adequate even thou the caregivers sings instead of speaking. Music activities were described to increase moods and socialization and are important part to increase a nice atmosphere. However, it does not increase cooperation in the way CS does. As interventions to facilitate caring situations with PWDs are needed, and CS has shown to be effective and should therefore be seen as an intervention not mixed up with traditional music activities. It is an important contribution in the education for staff in dementia care to include training in CS.

